# 新型分子靶向药物联合放疗在肺癌中的应用

**DOI:** 10.3779/j.issn.1009-3419.2010.08.19

**Published:** 2010-08-20

**Authors:** Mariano PROVENCIO, Antonio SANCHEZ, Pilar GARRIDO, Francisco VALCARCEL, 燕 丁, 军 李

**Affiliations:** 1 Department of Medical Oncology, Hospital Universitario Clínica Puerta de Hierro-Majadahonda; 2 Hospital Ramón y Cajal; 3 Department of Radiation Oncology, Hospital Universitario Clínica Puerta de Hierro-Majadahonda Madrid, Spain; 4 天津医科大学总医院，天津市肺癌研究所，天津市肺癌转移与肿瘤微环境重点实验室; 5 山东大学附属山东省立医院胸外科

**Keywords:** 贝伐珠单抗, 西妥昔单抗, 放化疗, 厄洛替尼, 吉非替尼, HSP90, Ⅲ期非小细胞肺癌

## Abstract

非小细胞肺癌（non-small cell lung cancer, NSCLC）约占肺癌总病例数的80%-85%，对于Ⅲ期患者来说，NSCLC约占肺癌总病例数的40%。不可切除Ⅲ期NSCLC的治疗为以铂类为基础的化疗联合胸部放疗。本文将综述正在研发中且有可能用于联合治疗的新型靶向制剂。其中最具前景的策略之一为表皮生长因子受体（epidermal growth factor receptor, EGFR）通路的抑制。放疗可激活EGFR信号，通过诱导细胞增殖并增强DNA修复而导致放疗抵抗。几项临床前模型研究表明西妥昔单抗与放疗联合具有协同效应。几项Ⅱ期试验评估了西妥昔单抗与放疗同步使用的安全性与疗效，结果喜人。吉非替尼对多种细胞系具有放疗增敏作用，其与放疗的联合已被试验用于不可切除Ⅲ期NSCLC的治疗。然而，放化疗后使用吉非替尼作为维持治疗的结果不容乐观。一项Ⅰ期试验评估了厄洛替尼与放化疗联合的疗效。放疗可通过损伤细胞膜、DNA以及微血管内皮细胞而诱导肿瘤死亡，而这反过来可增加促血管生成生长因子的产生。抗血管生长制剂可降低血管密度，但可改善肿瘤的含氧量。应用血管内皮生长因子受体（vascular endothelial growth factor receptor, VEGFR）抑制剂可通过阻断亚致死量辐射损伤的修复而增强放疗对人NSCLC的疗效。厄洛替尼、贝伐珠单抗与胸部放疗联合试验正在进行中。该三种药物联合治疗的新策略尚需制订。由于放疗可增强HSP90分子伴侣的功能从而引起肺癌细胞的放疗抵抗，此通路的阻断剂可通过抑制HIF-1α和VEGF的表达进而抑制肺癌细胞的生存和血管生成，因而可能用于减少放疗抵抗。在NSCLC和间皮瘤的临床前模型中，Aurora激酶抑制剂似乎对放疗具有增效作用。

## 前言

非小细胞肺癌（non-small cell lung cancer, NSCLC）约占肺癌总病例数的80%-85%，据估计2008年美国新诊断的肺癌病例超过215 000例。因诊断时大多数患者为晚期不可切除性肿瘤，预后较差。

不可切除Ⅲ期NSCLC患者约占肺癌全部病例数的40%。不可切除Ⅲ期NSCLC的标准治疗为同步采用以铂类为基础的化疗方案和胸部放疗。目前，尚无一种放化疗治疗方案可作为标准方案。化疗同步胸部放疗治疗可显著改善不可切除ⅡA期和ⅢB期肿瘤患者的生存期，为现有的治疗选择^[1, 2]^。

现有研究热点集中在新型制剂的研发以及治疗联合与新型制剂联合的评估。在此背景下，鉴别有潜力的生物靶标尤为重要，阻断这些靶标可影响多种下游信号级联。在本综述中，我们关注用于肺癌治疗的这些新型制剂以及有前景的与放疗联合的新的方案（表 1）^[3, 4]^。

**1:** 与放疗联合的主要新型药物 Major new agents in combination with radiation therapy

分类	治疗药物
EGFR通路西	妥昔单抗
	吉非替尼
	厄洛替尼
抗血管生成	ZD6474
	贝伐珠单抗
	镇静剂
	SU11657
mTOR通路	RAD001
	西罗莫司
热休克蛋白90抑制	格尔德霉素
组蛋白去乙酰化抑制剂	伏立诺他
Aurora激酶	PHA680632
	AZD1152
	ZM447439
Abbreviations: EGFR=epidermal growth factor receptor; mTOR=mammalian target of rapamycin Note: Reprinted with the permission from the copyright holder©CIG Media Group, LP 简写：EGFR=表皮生长因子受体；mTOR=哺乳类雷帕霉素靶蛋白 注：本表得到版权所有者©CIG Media Group, LP复制许可	

## 阻断表皮生长因子通路与放疗

最引人注目的通路之一为通过小分子（吉非替尼或厄洛替尼）或单克隆抗体（西妥昔单抗）联合放疗而抑制的表皮生长因子受体（epidermal growth factor receptor, EGFR）信号通路。

然而，在晚期患者中EGFR酪氨酸激酶抑制剂（tyrosine kinase inhibitor, TKI）与以铂类为基础的化疗联合并未产生任何生存获益^[5-8]^。

源自直接临床结果的有关TKIs与胸部放疗间关系的数据尚较少，但的确有数据提示在体外TKIs可增强化疗与放疗的作用。

## 临床前数据

西妥昔单抗是一种嵌合人-鼠单克隆抗体，可与EGFR结合，并可抑制无胸腺裸鼠体外及体内表达EGFR的癌细胞系的生长。放疗可激活EGFR信号，通过诱导细胞增殖并增强DNA修复而导致放疗抵抗。另外，其可引起G_1_期细胞的EGFR阻滞以及G_2_期细胞放疗时的EGFR阻滞，在理论上这意味着EGFR表达与放射治愈率之间存在反向关系，尤其在小鼠肿瘤中^[9]^。几项临床前模型研究表明西妥昔单抗与放疗联合具有协同效应^[10]^。

Raben等在一篇质量颇高的文章中通过免疫组化及流式细胞术评估了人NSCLC癌细胞系中EGFR的状态^[11]^。单独使用西妥昔单抗或其与放疗、化疗或放化疗联合处理NSCLC细胞系，以确定西妥昔单抗在荷NSCLC的无胸腺裸鼠中体外及体内的协同效应。仅在对西妥昔单抗敏感的NSCLC细胞系中观察到西妥昔单抗与放疗体外联合具有协同生长抑制效应。在对西妥昔单抗不敏感的NSCLC细胞系中并未观察到该协同效应。在表达EGFR、对西妥昔单抗敏感且伴有NSCLC细胞系异种移植物的裸鼠中，与任一制剂单独使用相比，西妥昔单抗与放疗联合可导致更为明显的肿瘤生长抑制的改善。

吉非替尼具有放疗增敏作用，这一点已在多种细胞系中得到证实^[12]^。研究表明，厄洛替尼可在多个水平（细胞周期阻滞、凋亡、诱导、加速的细胞再增殖以及DNA损伤修复）增强放疗的疗效^[13]^。

## 临床数据

### 西妥昔单抗

在肿瘤生长控制方面，体内研究显示西妥昔单抗与放化疗的三药联合治疗较两药联合无显著优势。一些近期报道的临床研究验证了该临床前观察。癌症和白血病B组（Cancer and Leukemia Group B, CALGB）30407（图 1）评估了使用培美曲塞、卡铂以及胸部放疗联合或不联合西妥昔单抗治疗不可切除Ⅲ期NSCLC患者的总生存期（overall survival, OS）。主要终点为生存超过18个月的患者所占的比例（两组均>55%）。中位随访19.5个月后，对照组的总有效率及中位生存期分别为73%和22.3个月，西妥昔单抗组则分别为71%和18.7个月。总之，在放疗和化疗方案中加入西妥昔单抗似乎并未带来进一步获益^[14]^。

**1:**
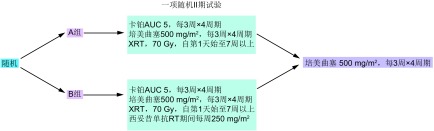
CALGB 30407：卡铂、培美曲塞以及放疗同步联合西妥昔单抗或不联合西妥昔单抗用于治疗不可切除Ⅲ期非小细胞肺癌 CALGB 30407: concurrent carboplatin, pemetrexed, and radiation therapy with or without cetuximab for unresectable stage Ⅲ non-small cell lung cancer

这些结果提示对于某些患者西妥昔单抗有益，但对于另外一些患者则有害，且西妥昔单抗的敏感性与EGFR表达水平并不明显相关，尽管其敏感性似乎需要一定程度的EGFR表达。在76%的表皮型肺癌及47%的腺癌中存在EGFR的过表达，因此理论上NSCLC是该联合方案的适应征。

然而，局限性晚期肺癌患者应用该治疗方法的安全性仍需研究验证。SCRTCH^[15]^研究旨在评估Ⅲ期NSCLC患者同时使用西妥昔单抗与根治性放疗的安全性。患者先接受以铂类为基础的诱导化疗，然后每周静脉注射西妥昔单抗（初始剂量，400 mg/m^2^；维持剂量，250 mg/m^2^）并同步进行放疗（64 Gy/32次/45天）。12例患者中的9例按计划完成了同步治疗，且未减量。3例患者未完成整套方案；1例患者在治疗中死于支气管肺炎。这些结果提示西妥昔单抗与根治性放疗同步使用的早期及晚期毒副作用均可接受。

NEAR试验^[16]^旨在评估西妥昔单抗与适型调强放疗（intensity-modulated radiation therapy, IMRT）局部照射联合治疗的毒性及可行性。

如上所述，该联合方案给人以希望。直至现在，最令人印象深刻的结果为美国肿瘤放射治疗组（Radiation Therapy Oncology Group）研究所获（RTOG 0324；图 2）^[17, 18]^。RTOG评估了西妥昔单抗（400 mg/m^2^，第1周的第1天）周剂量250 mg/m^2^直至治疗完成方案在不可切除NSCLC患者中的疗效。第2周，患者开始放化疗（63 Gy）联合每周卡铂曲线下面积（area under the curve, AUC）2、紫杉醇（45 mg/m^2^，6次），随后为2周期的卡铂（AUC=6）和紫杉醇（200 mg/m^2^）。主要终点为西妥昔单抗同步放化疗的安全性与依从性。治疗相关毒性可以接受；在87例可评价的患者中，17例患者出现4级血液系统毒性，7例患者出现3级食管炎。2年随访显示中位生存期为22.7个月，总生存率为49.3%，与之前在ⅢA/ⅢB期不可切除NSCLC患者中进行的RTOG研究相比，中位生存率及2年总生存率均较高。一项新近的小型研究报道了一类似的给药方案，每周的第1天加入顺铂6 mg/m^2^，放疗高达66 Gy。通过正电子发射断层扫描，该研究显示其代谢反应为50%，且毒性较低^[19]^。

**2:**
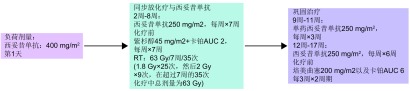
RTOG 032：一项西妥昔单抗联合放化疗治疗Ⅲ期非小细胞肺癌的Ⅱ期研究 RTOG 032: A phase Ⅱ study of cetuximab with chemoradiation therapy for stage Ⅲ non-small cell lung cancer

在另一项研究中，患者接受诱导化疗（多西紫杉醇/顺铂）随后同步进行西妥昔单抗治疗，研究显示放疗剂量为68 Gy是可行的。77%的患者接受了全程西妥昔单抗治疗，生存良好。中位生存期为17个月，3年生存率为33%^[20]^。

与单用放疗相比，放疗联合西妥昔单抗可显著延长局部晚期头颈部鳞状细胞肿瘤患者的局部控制时间和中位OS^[21]^。目前，得到证实的是，在其它肿瘤中，突变型或野生型K-*ras*的存在可影响其对西妥昔单抗的反应，在本综述发表之前这一点并不为人所知。很可能，对耐药机制的深入理解以及准确选择患者对改善肿瘤的预后有很大帮助。

### 吉非替尼

Stinchcomb及其同事研究了吉非替尼联合放疗在不可切除Ⅲ期NSCLC中的安全性^[22]^。高剂量三维适形胸部同步放疗的主要毒性作用为3级食管炎（19.5%）和心律失常（9.5%），但生存结果却令人失望，OS仅为9个月。

然而，值得提及的是一些间接结果，其可明显影响之后临床试验，如西南肿瘤组（Southwest Oncology Group, SWOG）Ⅲ期研究（S0023）的结果^[23]^。该试验旨在回顾性评估在非指定患者中吉非替尼在改善OS和无进展生存期方面的作用。采用顺铂、放化疗联合依托泊甙治疗后，无进展疾病的患者随后被随机分组接受安慰剂或吉非替尼作为维持治疗。一项包括573例患者的未计划的中期分析显示吉非替尼组较安慰剂组的OS短，吉非替尼组的中位生存期为23个月，安慰剂组为35个月（*P*=0.013）。如上所述，TKIs联合化疗的结果未见任何获益，但至少对患者生存无害。此类结果迫使我们寻找可能的原因。吉非替尼组的毒性似乎并不大，在头颈部肿瘤中其毒性与EGFR阻断及放疗亦无关。可能的是，之前的放疗治疗针对的是具有K-*ras*突变的细胞群，在该细胞群中TKI治疗的疗效较差^[24, 25]^。在正常的临床试验设计中难以避免此种类型的分子异质性^[26, 27]^。仅可能间接地推断可能的原因。

在CALGB 30106试验中，体能状态良好的患者接受诱导化疗随后同步放化疗，体能状态较差的患者接受诱导化疗随后仅实施放疗^[28]^。在放疗或放化疗期间或之后加用吉非替尼。两组耐受性均较好。“低风险组”中位OS为19个月，“高风险”患者为12个月；这些结果提示预后不良组的活性增强。

### 厄洛替尼

厄洛替尼（另一种TKI）的作用机制与吉非替尼相似。厄洛替尼与胸部放疗的治疗证据较吉非替尼少。简言之，尽管无额外毒性作用出现，结果并不鼓舞人心。Choong等实施的一项Ⅰ期研究旨在确定厄洛替尼联合2种标准放化疗治疗方案治疗NSCLC的最大耐受剂量，但中位生存时间却令人失望^[29]^。表皮生长因子受体免疫组化或荧光原位杂交（FISH）阳性患者在OS方面无显著差异。

研究表明，某些治疗手段可延长生存期，如同步化疗后使用厄洛替尼作为维持治疗，然而这些结果仅为非选用鳞癌组织学的早期研究^[30]^。

## 抗血管生成剂

人们感兴趣的另一方法为抗血管生成剂联合放疗^[31]^。肿瘤细胞可产生生长因子，其可刺激内皮细胞的增殖和迁移，并最终导致肿瘤组织内新生血管的形成。放疗可损伤细胞膜、DNA及肿瘤基质内的微血管内皮细胞，进而导致细胞死亡^[32, 33]^。作为对内皮损伤和缺氧的反应，肿瘤细胞可增加促血管生成生长因子的表达，如血管内皮生长因子（vascular endothelial growth factor, VEGF）和成纤维细胞生长因子^[34, 35]^。

因此，我们认为，抗血管生成治疗联合放疗可能会改善肿瘤病情。然而，在将荷瘤模型的治疗结论应用于患者时必须谨慎，因为根据荷瘤位置不同，基因表达可以有变化^[36]^。

应用ZD6474（一种强效的VEGFR2及EGFR赖氨酸激酶活性的口服抑制剂）可增强人NSCLC原位移植模型中放疗的疗效。在H441肺腺癌细胞的体外克隆形成细胞存活实验中，ZD6474治疗可消弱亚致死辐射损伤修复的能力，即使在肺癌细胞短期暴露于ZD6474之后。这些数据为针对人肺癌的生物靶向及传统治疗的临床试验提供了支持^[37]^。

有关贝伐单抗联合胸部放疗的研究尚较少。已报道的研究数据建议应慎用，且应采用临床对照试验。

一项Ⅱ期试验研究了小细胞肺癌患者使用卡铂、依立替康及贝伐珠单抗每两周10 mg/m^2^联合胸部放疗61 Gy的疗效^[38]^。在该项多中心、非随机、单组Ⅱ期临床试验研究中，29例患者中2例确诊患有严重的不良反应：气管支气管瘘。第3例死亡病例已报道，上呼吸消化道出血，死亡原因不明，疑为（但不确定）气管支气管瘘。所有的不良反应均发生在胸部放疗后使用贝伐珠单抗作为维持治疗的过程中，所有这些患者均患有3度食管炎。试验在早期即终止。在单独使用贝伐珠单抗和化疗或同步使用放疗治疗的肺癌和食管癌患者中气管支气管瘘的其他病例也有报道。根据这些作者的经验，气管支气管瘘的发生率为4.5%。局部组织损伤的增强和粘膜修复功能的受损与患者发生气管支气管瘘的机制相关。

与厄洛替尼、贝伐珠单抗及胸部放疗相关的另一项Ⅱ期研究已报道^[39]^。无死亡病例，3度食管炎的发生率为19.2%，2度食管炎的发生率为53.8%。尚缺乏明确的生存数据，但今年报道的数据较往年乐观^[40]^。因此，在这些药物与放疗联合时似乎应十分谨慎。患者的选择对于避免可能的致死性毒性非常重要。

沙立多胺联合化疗的抗血管生成活性亦已被研究。新近，一项有关不可手术的ⅢA期和ⅢB期NSCLC患者的研究结果被报道。研究比较了卡铂、紫杉醇及放疗与相同方案联合沙立多胺200 mg/d作为同步治疗24个月或直至疾病进展的疗效。277例患者被随机分组治疗，在发现无任何生存获益后该试验被终止。无沙立多胺组的中位生存期为15.3（12.4-20.2）个月，沙立多胺组则为16（14.4-18.3）个月^[41]^。

另外，不仅需要旨在寻找更佳疗效的研究，亦需要明确治疗的最佳次序及哪些药物应该与抗血管生成剂联合的研究。Huber等观察了血管生成抑制剂SU11657、培美曲塞及电离辐射等全部三种治疗方法同时服用的三药联合方案对人内皮细胞、A431人表皮样癌细胞的体外作用及对BALB/c小鼠中A431人肿瘤移植物的体内作用^[42]^。他们发现，无论体外还是体内，三药联合的抗内皮及抗肿瘤效应均优于单一药物及两药联合。研究还发现，SU11657治疗开始后给予放疗方案较放疗先于SU11657治疗更为有效。

尽管这些制剂在临床前研究中通常极为有效，但抗血管生成治疗与放疗在临床中的应用需要在适当的患者群体中实施合理的治疗方案以获得任何潜在获益^[43]^。

矛盾的是，抗血管生成剂可降低血管密度，但可改善肿瘤的含氧量。抗VEGFR治疗后肿瘤中pO_2_的浓度开始降低，但随后pO_2_稳步升高直至达到高于初始水平的水平。含氧量改善的一种可能的解释为血管的正常化，毛细血管弯曲度减弱。在这方面，在含氧量水平较高的组织中放疗更为有效^[44]^。

## 哺乳类雷帕霉素靶蛋白通路

磷脂酰肌醇3-激酶（phosphatidylinositol 3-kinase, PI3K）/AKT通路可被Ras或通路中组分的突变以及生长因子受体的Ras信号去调节作用所活化。对于暴露于可引起DNA损伤环境中的肿瘤细胞来说，Ras信号的激活可延长其生存。阻断EGFR/PI3K的活性可增强伴有K-*ras*突变的人肿瘤细胞的放疗敏感性^[45, 46]^。当被致癌性突变激活时，Ras的三种同分异构体H-*ras*、K-*ras*和N-*ras*均有助于放疗生存，这说明，致癌性Ras表达抑制可降低两种细胞系中的放疗生存^[47]^。

PI3K / Akt通路中的一个重要的下游效应器为哺乳类雷帕霉素靶蛋白（mammalian targen of rapamycin, mTOR）。mTOR通路在蛋白质合成及细胞生长的调节中发挥重要作用。mTOR最具特征性的功能为对蛋白质合成的调节^[48]^，其亦参与其它重要的细胞功能：如蛋白质的降解、蛋白质的稳定性、肌动蛋白细胞骨架的重构以及血管生成^[49]^。

mTOR的抑制可阻断放疗诱导的肿瘤细胞的应激反应，而应激反应可保护肿瘤微血管免受放疗损伤。在肿瘤细胞中，mTOR的抑制可预防放疗诱导的促血管生成生长因子的表达。内皮细胞似乎对mTOR抑制联合放疗最为敏感。因此，在体使用mTOR抑制剂RAD001和放疗导致的附加的肿瘤生长延迟或许取决于抗血管生成及抗血管的联合作用^[50]^。

一项Ⅰ期试验评估了标准三维适形胸部放疗（60 Gy）、每周顺铂（25 mg/m^2^ I.V.）与口服剂量逐渐增加的西罗莫司（sirolimus）联合的疗效。7例Ⅲ期肺癌患者被纳入该临床研究。接受2 mg/d西罗莫司治疗的4例患者中无一发生剂量限制性毒性。3例患者接受5 mg/d西罗莫司治疗，在该剂量水平1例患者发生3度吞咽困难的剂量限制性毒性。

该通路中的遗传变异可能调控临床预后且可被用于建立个体化治疗的模型^[51]^。

## 热休克蛋白90抑制

热休克蛋白（heat shock protein 90, Hsp90）作为分子伴侣在变性蛋白质，如AKT、HER2、Bsr-Abl、c-KIT、EGFR以及血小板源性生长因子受体（plateletderived growth factor receptor, PDGFR）-α的复性中发挥重要作用^[52]^。Hsp90抑制可导致化疗敏感及化疗抵抗小细胞肺癌细胞系中的大量细胞死亡。在临床上，格尔德霉素复合物最为成熟，毒性作用可控^[53]^。

## 组蛋白去乙酰化抑制剂

组蛋白去乙酰化抑制剂（histone deacetylase inhibitors, HDACs）在细胞运动中发挥作用且参与多种转录因子的调节。伏立诺他及其它HDACs在包括NSCLC的多种癌症中均取得了良好的疗效^[54]^。

ErbB3的表达或许可预测HSP90介导的肿瘤细胞对放疗的敏感性^[55]^。一项新近的研究为放疗增强的HSP90分子伴侣功能在升高肺癌细胞中缺氧诱导因子（hypoxia-inducible factor, HIF）-1α及VEGF的蛋白水平中发挥主要作用提供了第一手资料，HIF-1α及VEGF的蛋白水平的升高使肺癌细胞具有生存及血管生长的潜能^[56]^。放疗可通过两种机制的协同作用增加放疗抵抗肺癌细胞亚组中的HIF-1α蛋白水平：可刺激HIF-1α去重新合成的PI3K/Akt/mTOR的激活以及可导致HIF-1α蛋白稳定的Hsp90功能的激活。因此，阻断PI3K/Akt/mTOR和Hsp90功能的制剂可通过抑制HIF-1α和VEGF的表达从而抑制肺癌细胞的生存及血管生成，因而可能用于减少放疗抵抗。

## Aurora激酶

人丝氨酸/苏氨酸Aurora家族包括三个成员^[57]^，这三个成员与其它许多蛋白相互作用以调控有丝分裂过程中染色体的组装及分离。Aurora激酶在许多癌症类型中高度表达^[58, 59]^。Aurora A对有丝分裂纺锤体形成后中心体的正确分离以及早中期染色体的正确组织和分配非常重要。Aurora激酶A缺失时，有丝分裂纺锤体不能分离或折叠^[60]^。Aurora B是参与染色体分离和胞质分裂的一种染色体信使蛋白。Aurora激酶B通常在各种肿瘤中高水平表达，通常与Aurora A的表达一致，且表达水平与遗传不稳定性的增加以及临床预后不良相关^[61]^。目前大多数Aurora选择性小分子抑制剂正在临床前评估中^[62-66]^。

Aurora A的抑制联合放疗的疗效尚不明确。一项研究评估了通过PHA680632抑制Aurora A激酶对暴露于电离辐射（ionizing radiation, IR）后的细胞周期进展及肿瘤细胞存活的影响^[67]^。细胞周期分析显示暴露于PHA680632 24 h后DNA含量>4 N。当细胞同时接受辐射及PHA680632时，>4 N的DNA含量急剧下降。荷瘤小鼠的体内（p53-/- HCT116）研究显示，较单独IR相比，PHA680632-IR联合治疗可使肿瘤生长延迟增加。这些结果提示在癌细胞中PHA680632联合放疗可导致附加效应，尤其在p53缺失的细胞中，但无论体外还是体内PHA680632均非放疗增敏剂。

另一Aurora B激酶抑制剂AZD1152。AZD1152是一种喹唑啉前体药物，在血浆中转化为其活性代谢物AZD1152-HQPA，AZD1152-HQPA对Aurora B和C具有很高的亲和力。研究显示，IR前使用A ZD1152预处理细胞^[68]^可观察到附加效应。有趣的是，在p53缺失的细胞中可观察到更为明显的肿瘤细胞杀伤作用。荷瘤小鼠的体内研究证实IR联合AZD1152-IR较单独IR更可延迟肿瘤的生长。同时，这种效应在p53-/-HCT116及p53突变的异种移植物中更为明显。

这与进一步的临床治疗方案改善相关：在p53缺失的癌细胞中，Aurora B激酶抑制剂AZD1152在肿瘤对电离辐射的反应中的作用变弱。

Aurora B和survivin在间皮瘤中过表达。一项研究探讨了放疗是否可影响细胞中survivin及Aurora B的表达以及这些分子的抑制是如何影响放疗敏感性的。ZM447439及survivin反义寡核苷酸分别用以抑制survivin及Aurora B激酶。放射剂量为3 Gy时，可观察到survivin及Aurora B水平、Aurora B激酶活性以及G_2_/M期细胞的增加。放疗诱导的这些分子的上调可被survivin反义寡核苷酸及Aurora B小分子抑制剂ZM44739有效减弱。survivin和Aurora B的双重抑制可协同使间皮瘤细胞对放疗敏感，其剂量增强比为2.55。该治疗可导致放疗后多核细胞的形成增加，但并不增加裂解的capase 3的水平。survivin及Aurora B的抑制可导致放疗后间皮瘤细胞中的有丝分裂细胞停滞。这两种蛋白可能是恶性胸膜间皮瘤放射治疗的潜在治疗靶点^[69]^。

## 结语

靶向治疗联合同步放化疗治疗目前正在研究中。最有前景的治疗策略之一为EGFR通路的抑制。放射可激活EGFR信号，通过诱导细胞增殖并增强DNA修复而导致放疗抵抗。数项临床前模型研究表明西妥昔单抗联合放疗具有协同作用。一些Ⅱ期试验评估了西妥昔单抗与放疗同时使用的安全性与疗效，结果喜人。吉非替尼对细胞系具有放疗敏化作用，且其联合放疗用于不可切除Ⅲ期NSCLC的疗效正在研究中。

在临床前模型中血管生成治疗可增强放疗的疗效。抗血管生成制剂可降低血管密度，但可改善肿瘤的含氧量；因此，我们认为抗血管生成治疗联合放疗可改善肿瘤病情。新型药物联合同步放化疗已成为局限期晚期NSCLC的一种令人感兴趣的治疗选择。可以预测，进一步通过联合新型生物制剂有望改善生存。
